# 

**Published:** 1880-04

**Authors:** 


Vol. I. APRIL, 1880. No. 4.

### THE INDEPENDENT PRACTITIONER:

A MONTHLY JOURNAL,

DEVOTED TO

***MEDICAL, SURGICAL, OBSTETRICAL AND DENTAL SCIENCE.***

EDITED BY
HARVEY L. BYRD, A. M., M. D.,
AND
BASIL M. WILKERSON, D. D. S., M. D.

*"Altera poscit opem res, et conjurat amice."*

BALTIMORE:
THE PRACTITIONER PUBLISHING CO.,
B. M. WILKERSON, PROPRIETOR,
No. 68 North Charles Street.

$2.00 Per Annum in Advance. - - Single Copies, 25 Cents.

ENTERED AT THE POSTOFFICE AT BALTIMORE, MD., AS SECOND-CLASS MATTER.

